# Supplementary material for: Dysregulation of Tweak and Fn14 in skeletal muscle of spinal muscular atrophy mice
Source: Skelet Muscle. 2022 Jul 28;12:18. doi: 10.1186/s13395-022-00301-z (PMC9331072; doi:10.1186/s13395-022-00301-z)
Supplement: Supplementary file 1 — Additional file 1: Supplementary Figure 1. No overt dysregulation of Tweak and Fn14 in skeletal muscle of non-SMA hypomorphic Smn-depleted mice. qPCR analysis of Tweak (a), Fn14 (b), Pgc-1α (c), Mef2d (d), Glut-4 (e), HKII (f) and Klf15 (g) in triceps from post-natal day (P) 7 wild-type (WT), Smn2B/2B and Smn+/- mice. Normalized relative expressions are compared to WT. Data are mean ± SEM, n = 4-5 animals per experimental group, one-way ANOVA, Tukey’s multiple comparison test, * p < 0.05, ns = not significant. Supplementary Figure 2. Tweak and Fn14 are not dysregulated in denervated (nerve cut) muscles of pre-weaned mice. A sciatic nerve cut was performed on post-natal day (P) 7 WT FVB/N mice and both ipsilateral (nerve cut) and contralateral (control) TA muscles were harvested at P14. qPCR analysis of Tweak, Fn14, Pgc-1α, Mef2d, Glut-4, HKII and Klf15 in control and nerve cut TA muscles. Normalized relative expressions for each gene are compared to control muscle. Data are mean ± SEM, n = 7-11 animals per experimental group, two-way ANOVA, uncorrected Fisher’s LSD, ns = not significant. Supplementary Figure 3. Effect of varying Fc-TWEAK doses on disease progression in Smn‍-‍/‍-‍;SMN2 SMA mice. Smn‍-‍/‍-‍;SMN2 mice received daily subcutaneous injections of increasing doses of Fc-TWEAK (7.9, 15., 23.7 and 31.6 μg), starting at birth. a. Daily weights of untreated Smn-/-;SMN2 SMA mice and Smn-/-;SMN2 mice that received daily subcutaneous injections (starting at P0) of Fc-TWEAK (7.9, 15.8, 23.7 and 31.6 μg). Data are mean ± SEM, n = 5-10 animals per experimental group, two-way ANOVA, Sidak’s multiple comparison test. b. Survival curves of untreated Smn-/-;SMN2 SMA mice and Smn-/-;SMN2 mice that received daily subcutaneous injections (starting at P0) of Fc-TWEAK (7.9, 15.8, 23.7 and 31.6 μg). Data are presented as Kaplan-Meier survival curves, n = 5-10 animals per experimental group, Log-rank (Mantel-Cox). Supplementary Figure 4. Differential effect of Fc-TWEAK in skele [file Supplementary Figure 4.pdf › Supplementary Figure 4.pdf]

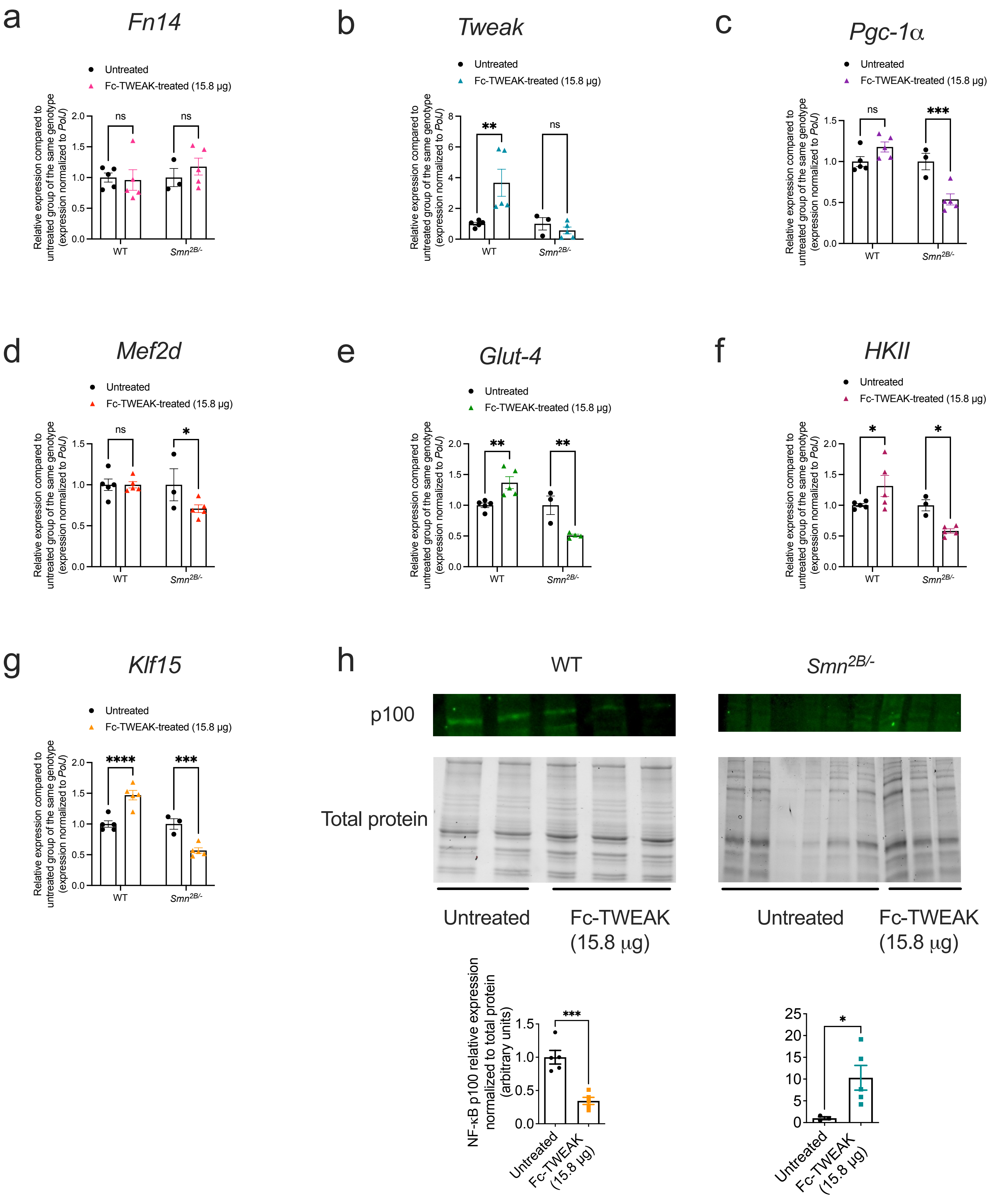

a
Fn14
Untreated
Fc-TWEAK-treated (15.8 μg)
Relative expression compared to untreated group of the same genotype (expression normalized to PolJ)
ns
ns
WT
Smn2B/-
b
Tweak
Untreated
Fc-TWEAK-treated (15.8 μg)
**
ns
c
Pgc-1α
Untreated
Fc-TWEAK-treated (15.8 μg)
ns
***
d
Mef2d
Untreated
Fc-TWEAK-treated (15.8 μg)
ns
*
e
Glut-4
Untreated
Fc-TWEAK-treated (15.8 μg)
**
**
f
HKII
Untreated
Fc-TWEAK-treated (15.8 μg)
*
*
g
Klf15
Untreated
Fc-TWEAK-treated (15.8 μg)
****
***
h
WT
Smn2B/-
p100
Total protein
Untreated
Fc-TWEAK (15.8 μg)
Untreated
Fc-TWEAK (15.8 μg)
NF-κB p100 relative expression normalized to total protein (arbitrary units)
***
*
Untreated
Fc-TWEAK (15.8 μg)
Untreated
Fc-TWEAK (15.8 μg)
